# Controlling the Hierarchical Morphology of ZIF-67 via Hydrothermal Synthesis: Insights into the Stability for Gas Separation

**DOI:** 10.1021/acs.langmuir.5c01142

**Published:** 2025-07-09

**Authors:** Paula S. Pacheco, Sônia Faria Zawadzki, Daniel Eiras

**Affiliations:** † Graduate Program in Materials Science and Engineering (PIPE/UFPR), 28122 Federal University of Paraná, Jardim das Américas, Curitiba, PR 81530-000, Brazil; ‡ Graduate Program in Chemistry (PPGQ/UFPR), Federal University of Paraná, Jardim das Américas, Curitiba, PR 81530-000, Brazil

## Abstract

This study focuses on optimizing the synthesis parameters of ZIF-67 by using the hydrothermal method and assessing the hydrothermal stability of the resulting hierarchical structures. The primary goal is to evaluate their structural integrity in aqueous environments, which is crucial for applications such as gas separation membranes. The optimization process involved dissolving 2-methylimidazole (MeIM) and the surfactant CTAB at controlled temperatures while vigorously stirring to promote micelle formation, followed by the addition of the metal precursor. This resulted in ZIF-67 particles with improved crystallinity and a more uniform morphology. Hydrothermal stability tests showed that ZIF-67 synthesized without CTAB was more resistant to structural degradation when exposed to water compared to the samples containing the surfactant. These results highlight the importance of synthesis conditions in enhancing the stability of ZIF-67, demonstrating its potential for gas separation applications, particularly CO_2_ capture.

## Graphical abstract

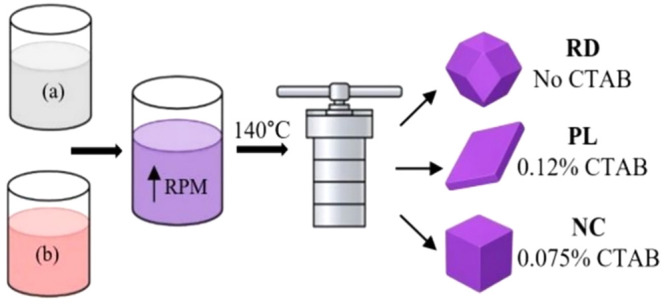

## Introduction

Metal–organic frameworks (MOFs) are a class of porous crystalline materials that have garnered significant attention due to their exceptional surface area, high porosity, and structural versatility.^1^ These features make MOFs highly applicable in diverse fields such as energy storage, catalysis, sensing, pollutant adsorption, and particularly gas separation.^2,3^

Among MOF subclasses, zeolitic imidazolate frameworks (ZIFs) stand out due to their zeolite-like topology, excellent chemical and thermal stability, and high selectivity for industrially relevant gases, such as CO_2_, CH_4_, and N_2_.^4^ The gas separation efficiency of these frameworks is directly linked to the pore aperture size, which, in molecular sieving, enables selective retention of larger gas molecules while smaller ones permeate the membrane.^3^

Surface porosity, pore volume, and particle size can be finely tuned by controlling synthesis parameters such as temperature, concentration, reaction time, and pH.^2,3^ ZIFs combine metal ions, typically in tetrahedral coordination, with imidazolate linkers that contribute with both flexibility and robustness to their structure.^5,6^ These characteristics make ZIFs, such as ZIF-8 (Zn) and ZIF-67 (Co), promising candidates for gas separation membranes, where precise molecular sieving is crucial.^6^

ZIF-67, composed of cobalt and 2-methylimidazolate, exhibits mechanical and chemical properties that make it particularly suitable for selective CO_2_ separation in complex gas mixtures and mixed matrix membrane production. This is attributed to its pore size (∼0.34 nm) and chemical affinity for CO_2_. The synthesis of ZIFs typically involves reticular synthesis methods such as hydrothermal, solvothermal, sonochemical, microwave-assisted, or room-temperature reactions, yielding highly ordered structures with robust metal–ligand bonds.^7^

However, achieving precise control over the morphology, size, and stability of ZIF-67 particles remains a significant technical challenge.^2,8,9^ Parameters such as temperature, reaction time, reagent concentration, pH, and the presence of modulating agents are critical for controlling crystal nucleation and growth.^10^ These parameters are closely tied to thermodynamic and kinetic conditions that determine whether the formation process is controlled by equilibrium or nonequilibrium.

Thermodynamically controlled products, typically obtained at higher temperatures, are more stable and energetically favorable.^10,11^ In contrast, kinetically controlled conditions, often associated with lower temperatures, may lead to the formation of metastable structures due to reduced activation energy barriers.^11,12^ Synthesis and crystallization methods play an essential role in tailoring ZIF properties, particularly morphology and crystal size.^10,13,14^

Three primary approaches have been explored to achieve such control: deprotonation regulation synthesis, which governs ligand deprotonation rates; coordination modulation synthesis, which employs additives to alter the coordination environment during crystallization; and surfactant modulation synthesis, which utilizes surfactants such as cetyltrimethylammonium bromide (CTAB) or polyvinylpyrrolidone (PVP) to modulate particle morphology and size.^12,15–20^

The pioneering work of Pan et al.^21^ demonstrated the use of CTAB in ZIF-8 synthesis to control particle morphology and size, revealing that varying CTAB concentrations resulted in significant differences in particle shape and size. Expanding on this concept, subsequent studies proposed morphological maps for hydrothermally synthesized ZIF-8 crystals.^22^

Hydrothermal stability is a critical factor for the practical application of ZIFs in mixed matrix membranes (MMMs). In aqueous environments, structural degradation may occur due to the hydrolysis of metal–ligand bonds, compromising both the functionality and selectivity of the membranes.^23,24^ This issue is particularly acute for ZIF-67, where the relatively weak bond between cobalt (Co^2+^) and methylimidazole makes it prone to disintegration in water. Additionally, the hydrophobic surface of ZIF-67 particles hampers their homogeneous dispersion, posing challenges for MMM fabrication.^25,26^

Residual surfactants, such as CTAB, can exacerbate these issues by limiting particle compatibility with polymer matrices and impairing long-term performance. While surfactants aid in initial morphology control, their residual presence necessitates innovative strategies to enhance hydrothermal resistance and ensure the viability of ZIF-67 for applications such as catalysis and gas separation.^27^

Efforts to address these limitations have included morphological modifications and surface engineering. For instance, Feng et al.^24^ demonstrated that ZIF-67 nanosheets exhibit enhanced hydrothermal stability in water due to the reduced surface energy and less exposed Co–N bonds. These findings suggest that tailoring the particle morphology and surface properties can effectively mitigate hydrothermal degradation.

This study investigates the influence of several synthesis parameters (mixing sequence, synthesis temperature, modulator concentration, type of ligand, mixing rotation speed, and premixing temperature), which were overlooked by previous research, on the formation of ZIF-67 particles with controlled morphologies, employing CTAB as a modulator. CTAB was chosen based on previous reported results that show that other surfactants such as CTAC are less efficient in the control of zeolitic imidazolate framework morphology. Moreover, CTAB is a suitable choice to investigate the influence of synthesis parameters on the morphology of ZIF-67 because previous work could be used as a frame of reference and comparison. Additionally, the hydrothermal stability, in the context of particle dispersion and mixed matrix membrane production, is evaluated in comparison with those synthesized via solvothermal methods. The goal is to identify conditions that favor the development of morphologically controlled and stable structures with potential applications in gas separation membranes. This work not only advances our understanding of ZIF-67 synthesis but also provides practical guidelines for the development of advanced materials for decarbonization and gas purification technologies.

## Experimental Section

### Materials

The synthesis of hierarchical ZIF-67 structures employed the following reagents: 2-methylimidazole (2-MeIM, MW = 82.10 g/mol, 99%), cobalt­(II) acetate tetrahydrate (Co­(OAc)_2_·4H_2_O, MW = 249.08 g/mol, 98%), and cetyltrimethylammonium bromide (CTAB, MW = 364.45 g/mol, 98%), all purchased from Sigma-Aldrich. Methanol (MW = 32.04 g/mol, 98%) was supplied by Êxodo, and ultrapure Milli-Q water was used for all experiments.

### Investigation of Hydrothermal Synthesis Parameters for Hierarchical ZIF-67 Structures

The hydrothermal synthesis of ZIF-67 was systematically studied by varying the order of reagent addition, synthesis temperature, and concentration of CTAB as a morphological modulator. Two precursor solutions were prepared: (a) 2-MeIM (65.36 mmol) dissolved in 32 mL of water and (b) Co­(OAc)_2_·4H_2_O (2.179 mmol) dissolved in 32 mL of water. Five experimental conditions were evaluated: (1) simultaneous mixing, in which the metal ligand, organic linker, and CTAB were simultaneously added to water at 25 °C; (2) premixing MeIM and CTAB (25 °C), in which 2-MeIM was premixed with CTAB in water at 25 °C before the addition of the metal ligand; (3) premixing MeIM and CTAB (100 °C), with the same procedure as condition 2, but with the mixture heated to 100 °C; (4) premixing metal ligand and CTAB (25 °C), in which the metal ligand was premixed with CTAB in water at 25 °C prior to the addition of 2-MeIM; and (5) premixing metal ligand and CTAB (100 °C), with the same procedure as condition 4, but with the mixture heated to 100 °C.

Additional parameters evaluated included stirring speed (450 or 1800 rpm) and reaction temperature (120 or 140 °C). Stoichiometric ratios were adjusted, with the MeIM:H_2_O molar ratio varied from 60 to 120. The optimized synthesis conditions identified were subsequently employed to prepare ZIF-67 particles for stability tests.

### Synthesis of ZIF-67 Particles for Stability Tests

The optimized hydrothermal synthesis conditions for ZIF-67 were based on a Zn^2+^:2-MeIM:H_2_O molar ratio of 1:32:1800, as reported by Yang et al.^22^ Two precursor solutions were prepared: (a) 2-MeIM (65.36 mmol) dissolved in 32 mL of deionized water, followed by the addition of CTAB, and (b) Co­(OAc)_2_·4H_2_O (2.179 mmol) dissolved in 32 mL of deionized water. Solution a was mechanically stirred at approximately 1800 rpm to promote the emulsification of CTAB, ensuring its uniform dispersion in an aqueous medium. After complete emulsification was achieved, solution b was slowly added to solution a while constant agitation was being maintained. The resulting mixture was further stirred until complete homogenization was achieved.

The final mixture was stirred for an additional 5 min. The CTAB concentration varied between 0.075% and 0.12% (w/w) to tailor the morphology of the resulting particles (nanocubes or plate-like structures). The reaction was conducted in a 150 mL autoclave containing 64 mL of a solution, and the mixture maintained at 140 °C for 24 h. After cooling to room temperature, the product was washed three times with methanol, followed by centrifugation at 10 000 rpm for 10 min using a Thermo Scientific Megafuge 11R centrifuge. The material was dried in an oven at 60 °C for 24 h. To remove any residual unreacted components, the particles were subjected to thermal treatment at 300 °C for 150 min under an inert atmosphere.

To evaluate hydrothermal stability, the synthesized particles were dispersed in an ethanol/water mixture (70:30, v/v) and sonicated at 30 °C for 30 min, followed by 2 h of rest. The samples were then centrifuged, dried, and analyzed. For comparison, an additional ZIF-67 sample was synthesized solvothermally using cobalt nitrate hexahydrate (Co­(NO_3_)_2_·6H_2_O), 2-MeIM, and methanol in a molar ratio of 1:8:1000. The synthesis of this sample was performed in a manner identical to that of the synthesis described in the previous study by the group.^28^ This sample was dried at 60 °C for 48 h. The conditions for stability tests were selected based on a common solvent mixture, and processing conditions were used to prepare Pebax mixed matrix membranes.

### Sample Nomenclature and Morphological Assignments

The synthesized ZIF-67 particles were designated based on the CTAB concentration and theoretical morphology, as detailed in Table 1. The CTAB concentration was selected based on the morphological map proposed by Pan et al., who synthesized ZIF-8 with different morphologies.

**Table 1:** Synthesized ZIF-67 Particles Labeled According to the CTAB Concentration and Their Theoretical Morphology

sample	synthesis type	CTAB concentration (% w/w)	theoretical morphology
RD	hydrothermal	none	rhombic dodecahedron
PL	hydrothermal	0.12	plate-like
NC	hydrothermal	0.075	nanocube
RD-1	solvothermal	none	rhombic dodecahedron

### Analysis

The morphology of ZIF-67 particles was characterized via scanning electron microscopy (SEM) using a JEOL JMS 6360-LV microscope with an acceleration voltage of 15 kV, a magnification of up to 75000×, and a resolution of 500 nm. Crystalline structure analysis was performed by using X-ray diffraction (XRD). Fourier-transform infrared spectroscopy (FTIR) measurements were carried out with a Bruker FTIR 70v spectrometer equipped with an attenuated total reflection (ATR) accessory.

The specific surface area of the particles was determined by using the Brunauer–Emmett–Teller (BET) method with nitrogen gas adsorption at 73 K, utilizing a Quantachrome NovaWin instrument. Thermogravimetric analysis (TGA) was conducted under a nitrogen atmosphere to evaluate the thermal treatment’s effectiveness in removing residual CTAB from the ZIF-67 particles.

This comprehensive characterization approach enabled the correlation among the synthesis conditions, morphological attributes, and structural integrity of ZIF-67, ensuring robust assessment of its properties for potential applications.

## Results and Discussion

### Investigation of Synthesis Parameters

#### Influence of the Mixing Sequence on the Synthesis of Hierarchical ZIF-67 Structures

Figure 1 presents the X-ray diffraction curves of ZIF-67 samples synthesized using defined CTAB ratios to achieve plate-like (PL) and nanocube (NC) morphologies. The XRD curves confirm the formation of the sodalite crystalline structure characteristic of ZIF-67, irrespective of the experimental mixing sequence or temperature conditions. However, significant variations in peak intensities reflect the direct impact of synthesis parameters on the material’s crystalline organization.

**Figure 1:**
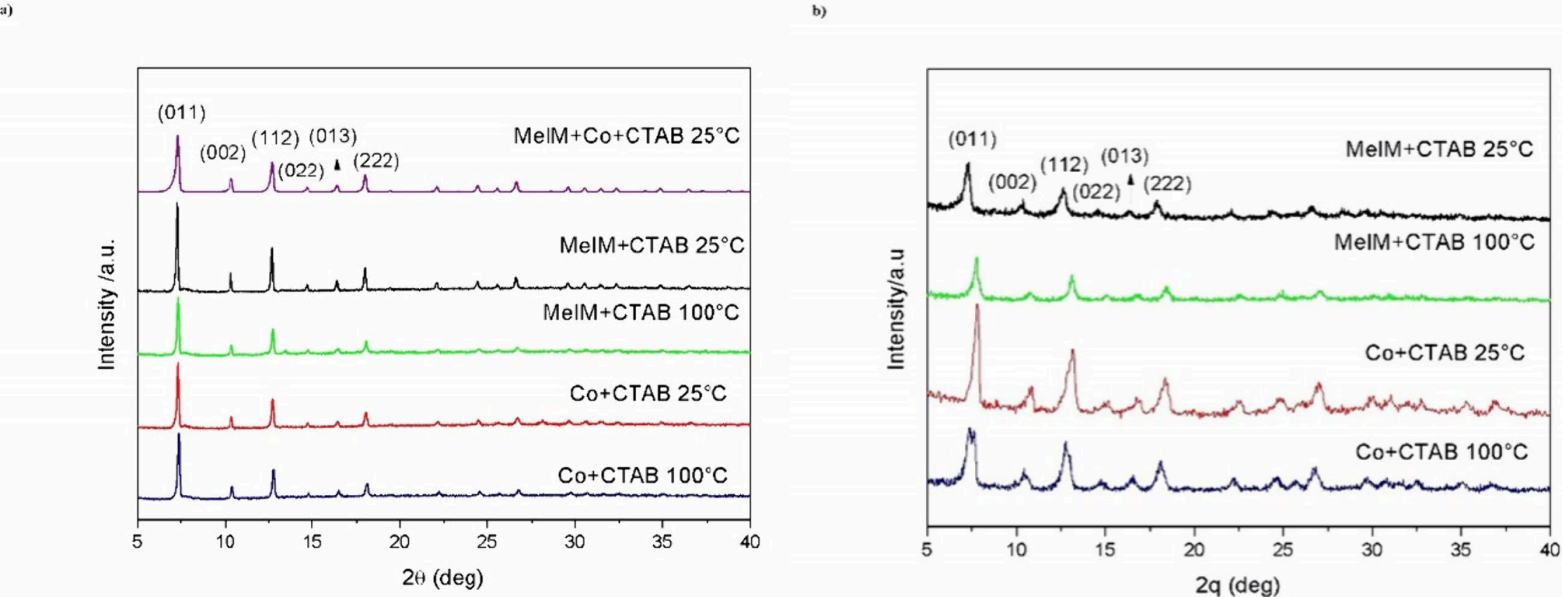
XRD curves of ZIF-67 synthesized with defined CTAB ratios for producing (a) plate morphology (PL) and (b) Nanocube morphology (NC).

For plate-like morphology, a pronounced increase in the intensity of the peak corresponding to the (011) plane was observed under the MeIM/CTAB mixing condition at 25 °C, indicating a more ordered and crystalline structure. This suggests that lower temperatures, coupled with the preinteraction between the modulator (CTAB) and the organic ligand, create a favorable reaction environment for forming highly organized structures.^29^

In general, synthesis methods using preheated solvents in autoclaves (50–200 °C) with reaction times of 12–72 h involve hydrolysis, coordination, and deprotonation processes. These interconnected mechanisms allow precise control over the final material properties.^29,30^ Although all conditions resulted in ZIF-67 formation, the XRD curves of cubic samples revealed broader peaks and interference, likely associated with unreacted materials, impurities, or undesired crystalline phases. These results highlight the system’s sensitivity to synthesis parameters such as mixing sequence and temperature control.

The addition of surfactants to precursor solutions plays a pivotal role in the synthesis. CTAB, a surfactant with hydrophilic and hydrophobic properties, acts as a protective agent, modulating particle arrangement during cobalt crystal formation.^30^ Previous studies have suggested that surfactants like triethylamine (TEA) can deprotonate imidazole-based ligands, preventing the formation of undesired metal hydroxides.^18,31^ This is particularly relevant in ZIF synthesis, where the methyl group in MeIM acts as a structural agent, replacing the need for organic solvents and initiating nucleation to prevent hydroxide formation.

Additionally, peak broadening in the diffractograms indicates microstructural imperfections, including residual stresses, crystalline distortions, and local disorder, phenomena linked to nonideal nucleation and growth conditions.

CTAB plays a crucial role in controlling the morphology during synthesis. Its amphiphilic properties facilitate particle interaction and help regulate ZIF-67 crystal formation. For ZIF-67, the methyl group in MeIM is critical in replacing complex organic solvents, initiating nucleation and preventing metal hydroxide species formation. Impurities, such as zinc or cobalt hydroxides or nitrates, are frequently observed in aqueous syntheses due to competition between OH^–^ ions and MeIM ligands for binding to the metal ion. To minimize these effects, a significant excess of MeIM is required, as reported by Fan et al.^32^

In methanol, nucleation occurs without the need for additional bases, as the solvent naturally promotes metal–ligand complex formation. Gross et al.^31^ emphasized that aqueous systems require substantially higher surfactant levels and ligand amounts than methanol-based systems, underscoring the solvent’s importance in nucleation. For CTAB, its dual role as a morphological modulator and deprotonating agent allows for more defined structures with less reagent consumption.

The initial interaction between CTAB and MeIM was critical for crystal formation in both plate and nanocube morphologies. For plates, SEM micrographs (Figure 2) revealed structures closer to the desired pattern under both temperature conditions (25 and 100 °C), indicating that CTAB effectively adjusted particle growth.

**Figure 2:**
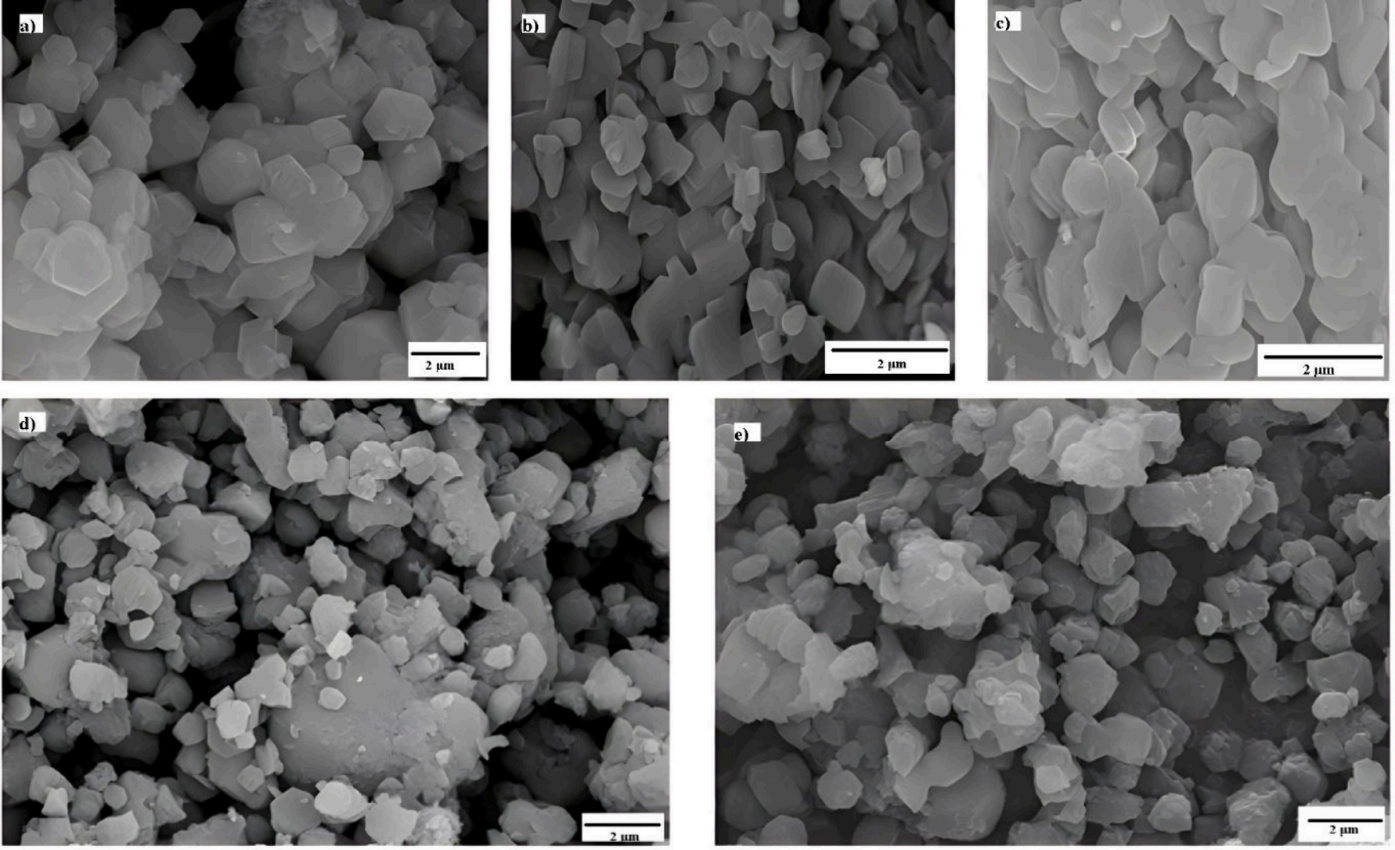
SEM images of plate-like ZIF-67 synthesized with different mixing sequences: (a) MeIM, Co, and CTAB at 25 °C, (b) MeIM and CTAB at 25 °C, (c) MeIM and CTAB at 100 °C, (d) Co and CTAB at 25 °C, and (e) Co and CTAB at 100 °C.

For nanocubes, Figure 3 shows more well-defined facets when CTAB is dissolved in MeIM before synthesis. However, all four evaluated synthesis conditions afford particles of varying sizes and irregular morphologies. These findings align with XRD analysis, suggesting inconsistencies in experimental parameters such as reagent addition sequence or ligand deprotonation rate.

**Figure 3:**
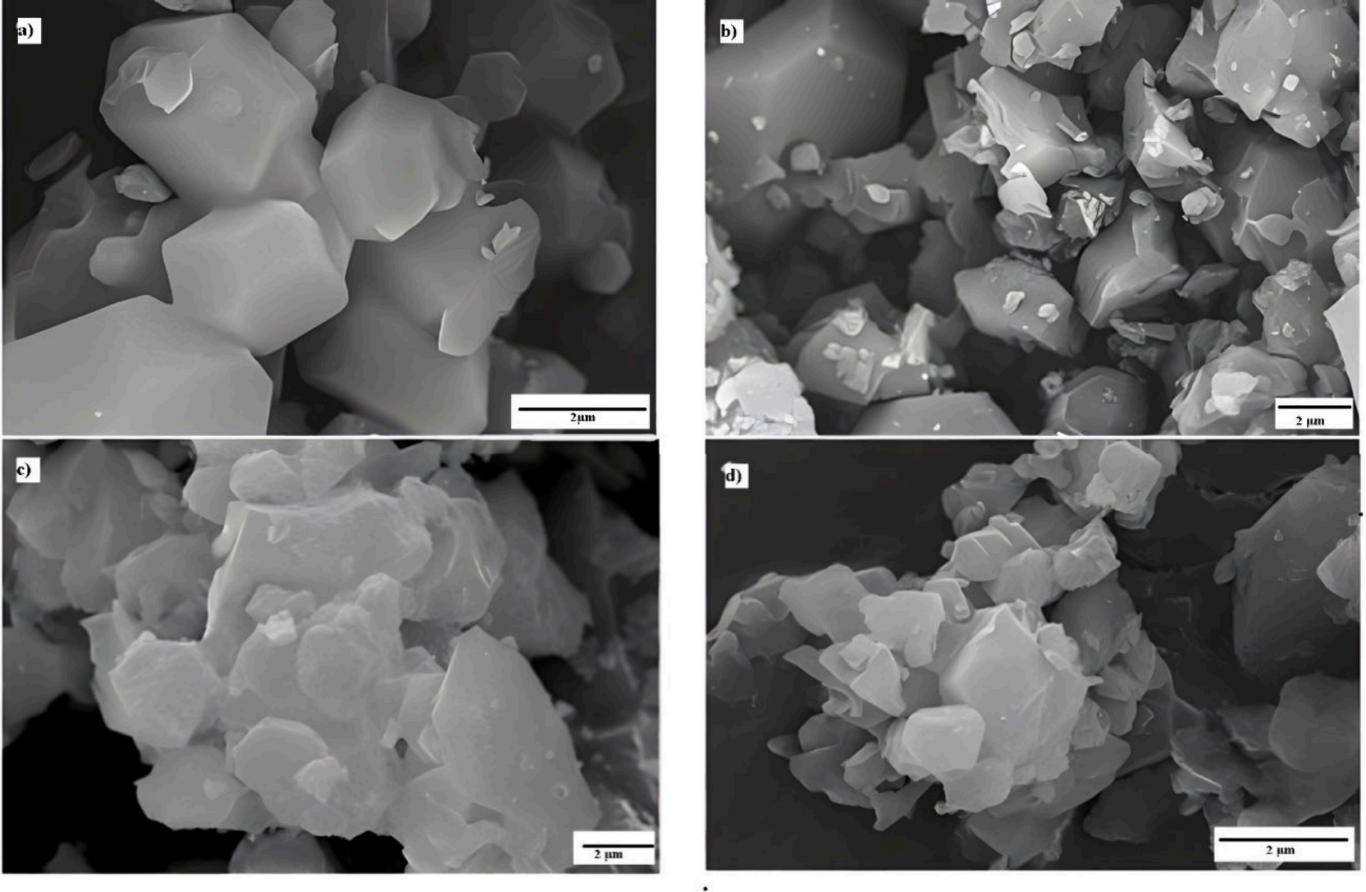
SEM images of nanocube-like ZIF-67 synthesized with different mixing sequences: (a) MeIM and CTAB at 25 °C, (b) MeIM and CTAB at 100 °C, (c) Co and CTAB at 25 °C, and (d) Co and CTAB at 100 °C.

These results emphasize the importance of stricter kinetic control during synthesis and adjustments to experimental conditions to achieve a more homogeneous and controlled ZIF-67 morphology. Future studies may explore variations in reaction time, reagent proportions, and thermal profiles as strategies to optimize particle formation with uniform morphology and size.^14,18,31^

#### Influence of Stirring Speed and Temperature on ZIF-67 Synthesis

This study evaluated the impact of the stirring speed and temperature on the synthesis of ZIF-67. The XRD patterns shown in Figure 4 confirm that vigorous stirring led to the successful formation of crystalline ZIF-67, regardless of the temperature. Additionally, the XRD peak intensities were higher for samples synthesized at 140 °C compared to 120 °C, indicating that higher temperatures promote better crystallinity.

**Figure 4:**
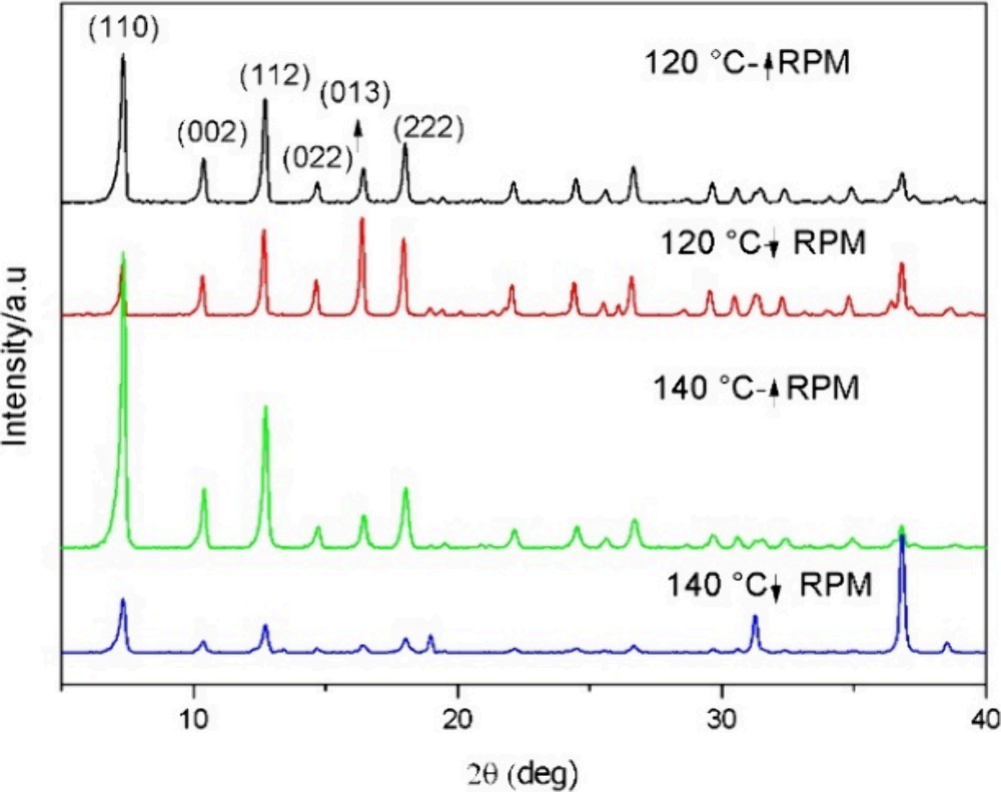
XRD curves of ZIF-67 obtained under different stirring speeds and synthesis temperatures.

Previous studies, such as those by Sun et al.,^33^ demonstrated that reverse microemulsions (water-in-oil systems) provide an effective environment for synthesizing monodisperse ZIF-67 nanoparticles. In this method, CTAB acts as a surfactant while 1-hexanol and heptane serve as the cosurfactant and oil phase, respectively. Micelles formed in these systems act as nanoreactors, enabling precise control over the crystal size and morphology. Furthermore, the nanostructures synthesized using this method exhibited significantly larger specific surface areas and pore volumes than those obtained in conventional aqueous media.^29,33^

Although thermodynamically unstable, emulsions play a crucial role in nanocrystal synthesis by concentrating less hydrophilic ligands, such as MeIM, within the micelles. This mechanism protects metal–ligand intermediates from interacting with OH^–^ ions, thereby preventing the formation of impurities and promoting selective nucleation. Konno et al.^34^ observed that, in the absence of micelles, particle size reduction was not achieved, underscoring the role of surfactants in controlling crystal growth. Moreover, the presence of micelles in the solution ensures a homogeneous distribution of reagents and provides greater control over crystal growth.^30,34^

When a surfactant is present in an aqueous mixture subjected to vigorous stirring, micelles form through hydrophilic and hydrophobic interactions. In these micelles, MeIM molecules and Co–MeIM intermediates, which are less hydrophilic, are concentrated, while OH^–^ ions, which are more hydrophilic, are predominantly excluded. This exclusion protects Co–MeIM intermediates from interacting with OH^–^ ions, preventing the formation of undesirable metallic hydroxides and resulting in the formation of pure ZIF-67 nanocrystals.^32^

The crystal size distribution is a critical parameter in nanocrystal synthesis. Although Cravillion^18^ reported that unstirred solutions yield crystals with narrower size distributions, microscopy images (Figure 5) from this study indicate that vigorous stirring can produce crystals with more homogeneous sizes, especially in plate-like morphologies. This discrepancy can be attributed to specific synthesis conditions, such as surfactant use and emulsion control, which favor more uniform nucleation during the initial stages.

**Figure 5:**
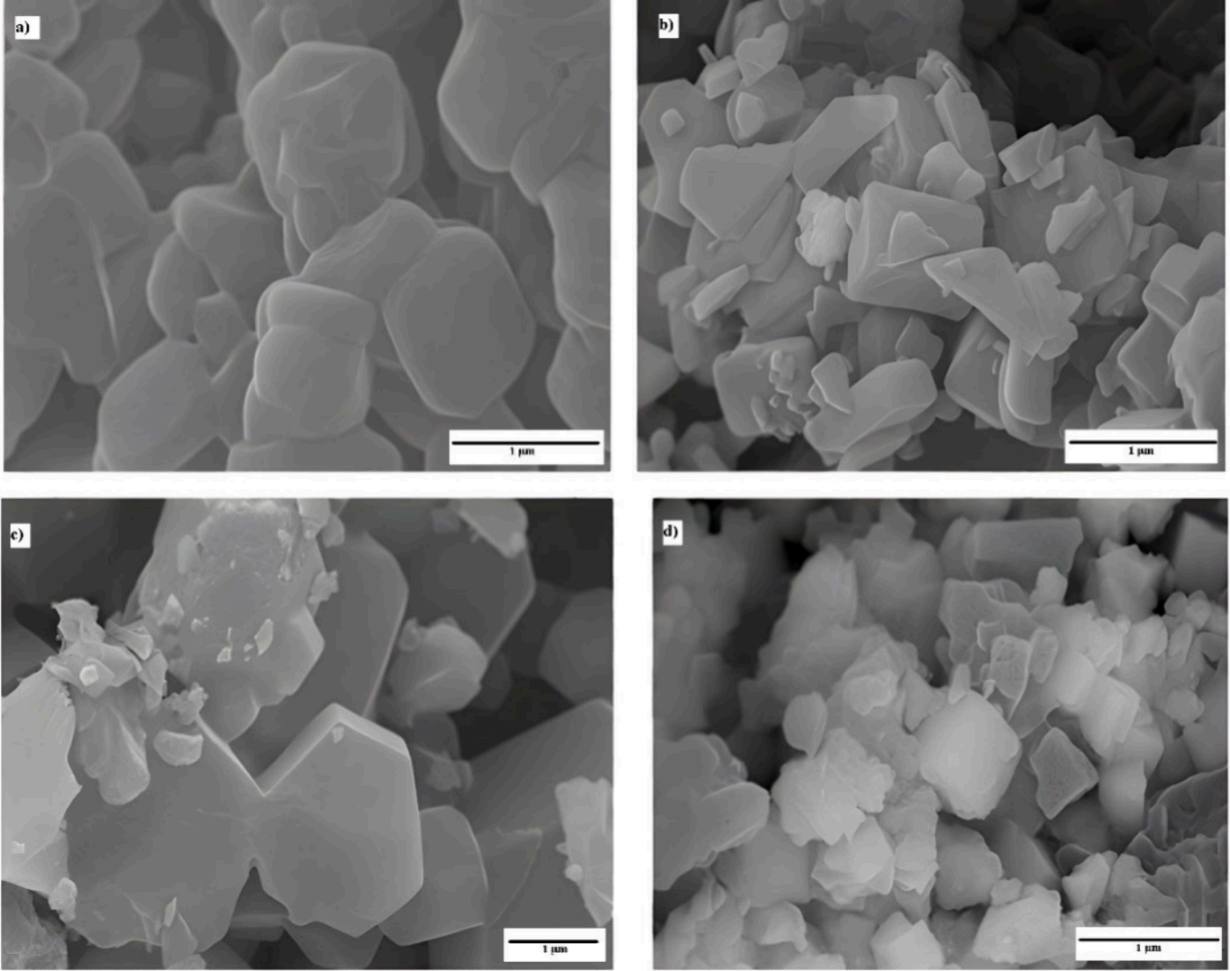
Scanning electron microscopy of ZIF-67 synthesized at different stirring speeds and temperatures: (a) 120 °C and a high stirring speed, (b) 120 °C and a low stirring speed, (c) 140 °C and a high stirring speed, and (d) 140 °C and a low stirring speed.

Regardless of the synthesis temperature, mixtures subjected to vigorous stirring and emulsion formation yielded particles with higher crystallinity, more regular morphologies, and more homogeneous size distributions than those synthesized under weak stirring conditions. These results highlight the critical role of the initial emulsion and micelle formation in controlling directional particle growth.

Micelle formation appears to act as a stabilizing mechanism during the nucleation and growth processes, concentrating ligands and metal intermediates in an environment conducive to the formation of well-defined crystals. Although vigorous stirring may increase the crystal size dispersion due to secondary nucleation induced by turbulence, the results suggest that micelle-mediated control is predominant, yielding particles with more uniform sizes and more stable structural characteristics.

Thermogravimetric analysis, shown in Figure 6, further confirmed the efficiency of ZIF-67 production under different stirring and temperature conditions. TGA curves obtained under a synthetic air atmosphere revealed a single weight loss stage for samples synthesized under vigorous stirring, indicating primary structural degradation between 340 and 430 °C. Under these conditions, weight losses of 57% and 51% were observed for samples synthesized at 140 and 120 °C, respectively.

**Figure 6:**
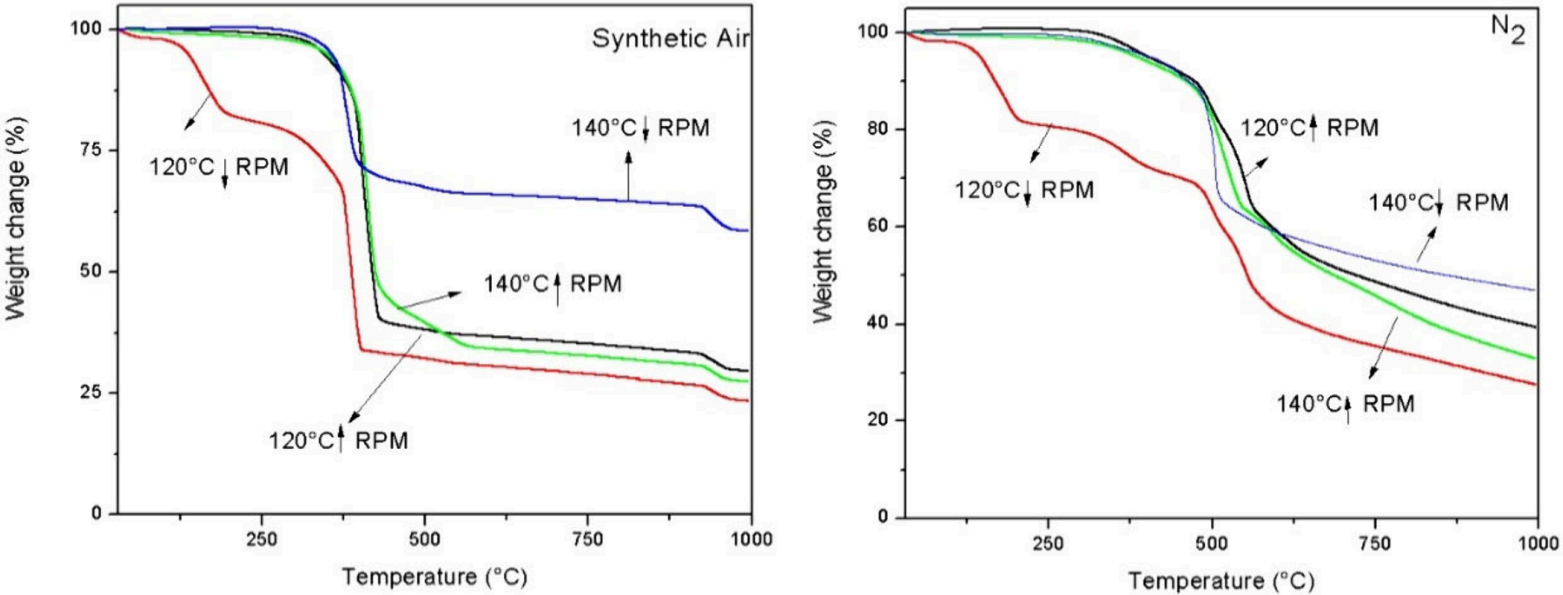
Thermogravimetric analysis (TGA) of ZIF-67 synthesized at different stirring speeds and temperatures: analysis in synthetic air (left) and analysis in nitrogen (right).

Conversely, the TGA curve for synthesis at 140 °C under low stirring speed also exhibited a single weight loss stage, starting at 310 °C, with 70% of the initial weight remaining. However, samples synthesized at 120 °C with a low stirring speed displayed two distinct weight loss stages. The first stage, beginning at 100 °C, was attributed to MeIM decomposition, resulting in a 16% weight loss. The second stage, corresponding to the degradation of the crystalline structure, occurred at 305 °C and resulted in a total weight loss of 45%.^22,35^

Additional XRD and scanning electron microscopy (SEM) analyses, provided in Figures S1 and S2, revealed that for a fixed MeIM:H_2_O molar ratio of 120, none of the tested conditions led to the formation of the desired ZIF-67 phase. Microscopic images showed irregular structures lacking well-defined crystal facets. These findings contrast with those of Yang et al.,^22^ who reported the successful formation of plate-like particles when varying the MeIM:H_2_O molar ratio between 40 and 200. Additionally, their study noted that the plate size decreased with an increase in H_2_O:MeIM ratios and increased with CTAB concentration.

Under the synthesis parameters adopted here, initially formed micelles played a crucial role as microreactors, creating favorable conditions for the synthesis. However, the coalescence of microemulsions upon transferring the substance to a heated reactor at higher temperatures led to less controlled crystal formation. Temperature, stirring, and surfactant (CTAB) significantly influenced the final crystallinity and morphology of the particles.^30,36^

A high MeIM concentration in aqueous media was necessary to prevent the formation of metallic hydroxides. The microemulsion promoted rapid interaction between MeIM and Co^2+^ ions, forming Co–MeIM intermediates that favored nucleation and resulted in more regular and well-defined crystals.^30–32^ CTAB acted as a protective agent, preventing the formation of undesirable impurities and contributing to the purity of ZIF-67 crystals.^24,32^

These results emphasize the importance of stringent control over synthesis conditions, particularly the use of surfactants and the selection of reaction temperatures, to ensure ZIF-67 with the desired characteristics.

### Synthesis of ZIF-67 Particles and Study of Hydrothermal Stability

X-ray diffraction analysis of the particles (Figure 7) revealed that the RD, RD-1, and PL samples exhibited a pure phase of ZIF-67, with the main peaks corresponding to the (011), (002), (112), and (222) planes for ZIF-67. Although the intensities of these peaks varied, they were well-defined, indicating high crystallinity. However, the XRD curve for the NC particle exhibited low crystallinity, suggesting that the desired structure was not achieved during synthesis, as confirmed later by scanning electron microscopy.^11,37–39^

**Figure 7:**
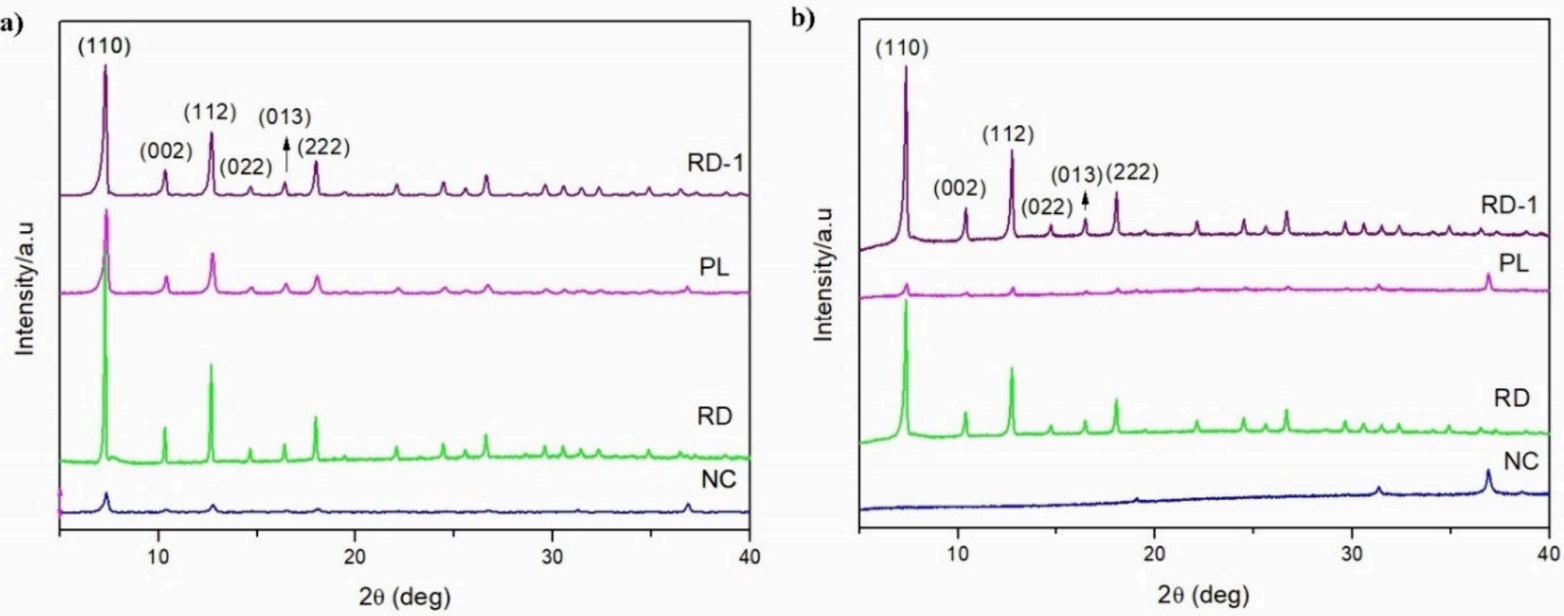
XRD curves of the samples (a) before and (b) after the stability test.

After the stability test, the XRD analyses showed that the crystal structures of RD-1 and RD remained stable, with no significant changes observed in the XRD curves. In contrast, instability was noted in the surfactant-added particles, as made evident by a comparison of the two XRD analyses. Despite the initial XRD curve of the nanocubes suggesting that the desired structure was not formed after contact with water, peaks that initially had low intensity were not detected in the poststability test analysis, indicating potential particle degradation. This pattern was also observed in the morphologies of the plates. Notably, the intensity of the peak at 37° increased in the XRD curves after the stability test for the surfactant-synthesized particles. Overall, for ZIF-67, the multiple reflections displayed in the curves are characteristic of the cobalt structure.^40^

FTIR spectra of the synthesized crystals are shown in Figure 8. For RD-1, spectral bands observed at 689 and 764 cm^–1^ are attributed to the out-of-plane bending of the MeIM ring, while peaks around 990 and 1294 cm^–1^ are assigned to in-plane bending. The intense peak observed at 1483 cm^–1^ corresponds to stretching of the MeIM aromatic ring, and the bands at 1583 and 1665 cm^–1^ are related to N–H vibrations and N–H bending in the MeIM molecule, respectively. Finally, the bands at 2930 and 3138 cm^–1^ are attributed to the aliphatic and aromatic C–H stretching of MeIM, respectively. For RD, bands similar to those of RD-1 were observed for both pre- and poststability test samples, but a band shift between 2406 and 3554 cm^–1^ before the stability test was evident.^20,41–43^

**Figure 8:**
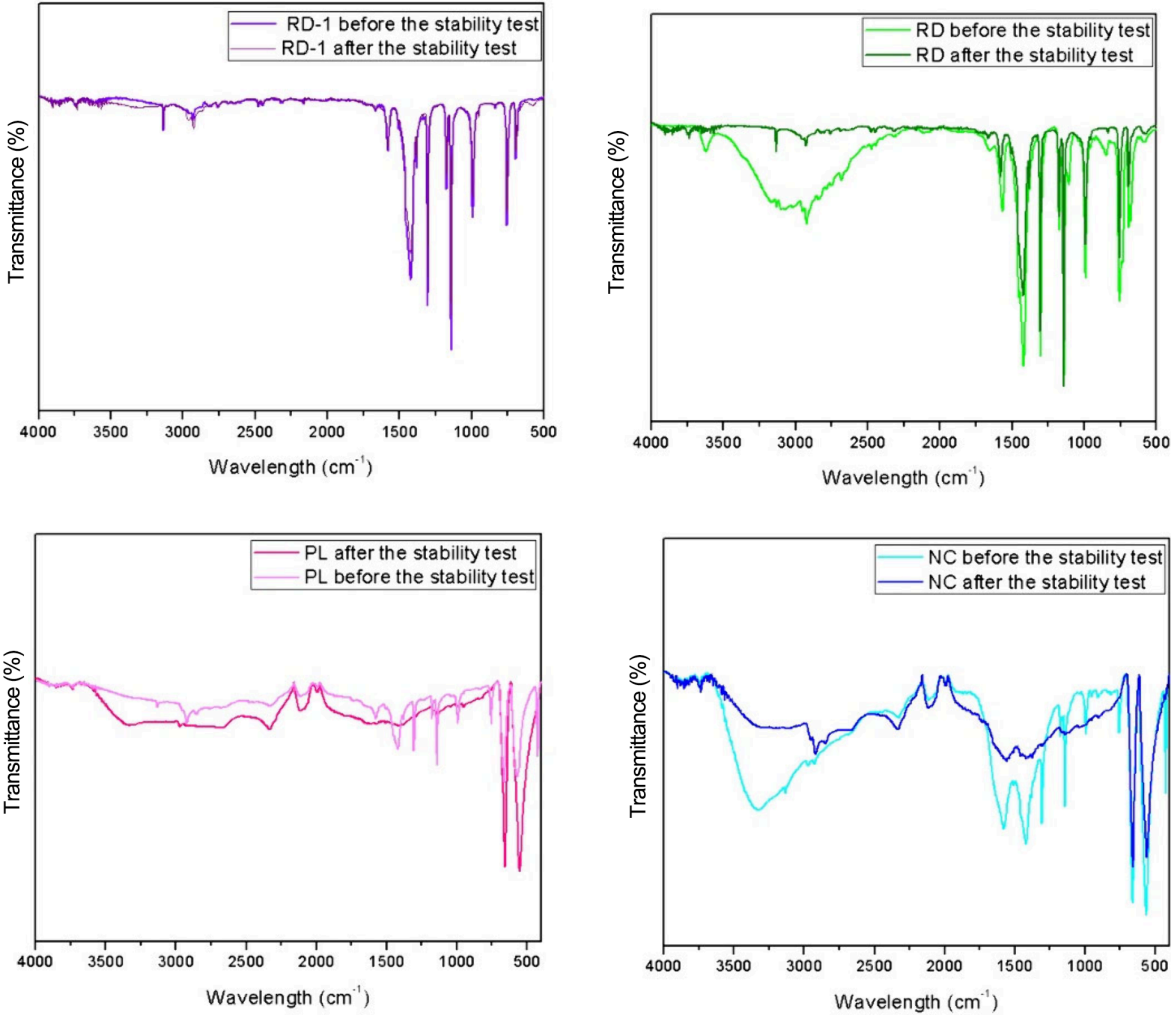
FTIR spectra of the samples before and after the stability test.

The appearance of this broad band overlapping with the CH-related bands is attributed to the stretching vibration of OH in the solvent, explaining the disappearance of this band after the stability test.^20,41^ A similar behavior was observed for the PL and NC morphologies, leading to the conclusion that the presence of this band is related to the hydrothermal synthesis method, where water was used as the solvent instead of methanol, as in the RD-1 synthesis. Previous studies also investigated the stability of ZIF-67 under different conditions. Feng et al.^24^ conducted a similar procedure to investigate the stability of the synthesized structures. They concluded that both nanoparticle and microparticle structures of ZIF-67 were damaged, while the modified nanosheet structure remained stable.

The results of this study, from both X-ray diffraction and Fourier-transform infrared spectroscopy, point to the stability of ZIF-67 synthesized via the solvothermal method when it is exposed to water. For the RD particle, the FTIR spectrum revealed the presence of the hydroxyl group, identified by a band at 3026 cm^–1^; however, after the stability test, this feature was not detected, and no significant changes were observed in the other bands.^44,45^ With regard to the surfactant-containing particles, both still exhibited CTAB in their structure. According to FTIR analysis conducted by Yang et al.,^22^ the characteristic peaks of CTAB were detected in all of the formed morphologies. However, the authors observed that after thermal treatment no band related to the presence of CTAB was identified, suggesting its complete removal during this procedure.

Thermogravimetric analysis of the samples after the stability test was conducted in synthetic air and N_2_ and is represented in Figure 9. For the ZIFs, the first stage of gradual weight loss corresponds to the removal of guest molecules and nonreactive species. In synthetic air, the decomposition temperatures for MeIM and CTAB range from 150 to 200 °C and from 230 to 300 °C, respectively.^22,33^

**Figure 9:**
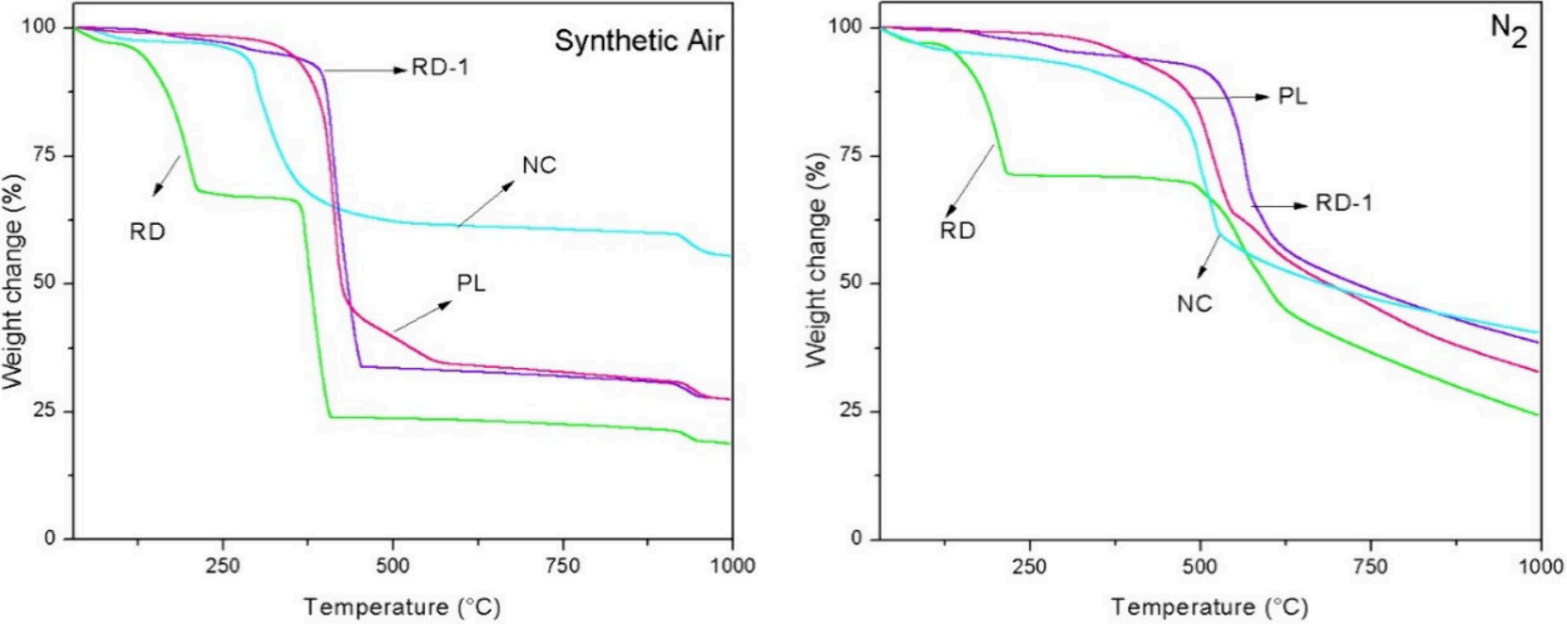
Thermogravimetric analysis of the samples after the stability test.

For the RD-1 sample, an initial slight weight loss is observed under both conditions. In synthetic air, weight loss begins at 330 °C and ends at 460 °C, resulting in approximately 58% total loss. In N_2_, the primary weight loss starts at 505 °C, reaching 44%, with a total loss occurring by 630 °C. The onset and final temperatures for mass loss are presented in Tables S1 and S2. For the RD sample, two distinct weight loss stages are identified under both conditions. In synthetic air, the first stage starts at 120 °C, corresponding to 28% weight loss, which can be attributed to MeIM decomposition.^46^ The second stage, related to the degradation of the ZIF structure, occurs at 350 °C and continues until 500 °C. The onset and final temperatures for these stages are detailed in the Supporting Information.

The plate morphology (PL) and nanocube morphology (NC) exhibit distinct thermal profiles. For the plate morphology (PL), a single weight loss occurs at approximately 350 °C in air and 323 °C in N_2_, resulting in total losses of 35% and 63%, respectively. For the nanocube morphology (NC), weight losses of approximately 64% in air and 59% in N_2_ are observed. The precise onset and final temperatures for these mass losses are provided in the Supporting Information. The small initial weight loss observed for the nanocubes, starting at 64 °C in air and 32 °C in N_2_, is attributed to the release of incorporated MeIM molecules.^22–24^ As described by Ribeiro et al.^42^ the initial weight loss in ZIF-67 corresponds to the removal of water and organic ligand molecules trapped within the structure’s pores.

The degradation onset temperature for the nanocube (NC) sample was 64 °C in air and 32 °C in N_2_, indicating lower thermal stability compared to the other morphologies. This result aligns with the findings from the previously discussed XRD curves. Importantly, the plate (PL) and nanocube (NC) morphologies did not show significant weight losses related to the presence of CTAB in the particles.^22^ This observation is expected, given the low surfactant content ranging between 0.08% and 0.12% by weight.

Surface area analysis, via the BET method, of the ZIF-67 particles synthesized (Table 2) revealed large surface areas for the RD-1 and RD samples. Before the test, the surface area of the RD-1 sample was 1414.345 m^2^/g, increasing to 2397 m^2^/g after the test. For the RD sample, the initial surface area was 986.736 m^2^/g, increasing to 1370.158 m^2^/g afterward.

**Table 2:** Brunauer–Emmett–Teller (BET) Surface Area Analysis of the Synthesized Particles

sample	metal:ligand:solvent	surfactant (wt %)	metal source	surface area before the test (m^2^/g)	surface area after the test (m^2^/g)
RD-1	1:8:1000/methanol	–	Co(NO_3_)_2_	1414.345	2397.713
RD	1:32:1800/water	–	Co(OAc)_2_	986.736	1370.158
PL	1:32:1800/water	0.12	Co(OAc)_2_	794.545	594.5450
NC	1:32:1800/water	0.075	Co(OAc)_2_	333.750	0.000

These results corroborate the observations obtained from XRD and FTIR analyses, indicating the preservation of the crystalline structure. The increase in surface area in both samples after exposure to a water/ethanol mixture is unexpected and could be associated with the removal of undissolved precursors from the ZIF-67 surface, such as methylimidazole. A comparison between the similarly polyhedral morphologies of RD-1 and RD suggested a reduction in the surface area for materials synthesized in water. This is because the size of the ZIF particles cannot be well-controlled when synthesized with water as a solvent, as the growth kinetics in the aqueous phase is generally faster than in methanol.^31,33,34^

Studies have shown that the synthesis of zeolitic imidazolate frameworks (ZIFs) at different temperatures results in surface areas approximately 50–70% smaller than those obtained at room temperature in aqueous solutions. The metal source and molar ratio of the reagents also significantly influence the properties of the synthesized materials. An optimal concentration ratio between the reagents is necessary to achieve larger surface areas in aqueous solutions, requiring a higher proportion of MeIM. However, an excess of unreacted MeIM may fill the external porosity and cavities, leading to a reduction in the crystal surface area. The choice of the metal source can also substantially affect the properties of the synthesized materials. Additionally, particles produced through microemulsions in aqueous solutions can exhibit surface areas comparable to or even larger than those obtained via a methanol-based synthesis. However, in this study, particles synthesized with a surfactant demonstrated surface areas of less than 1000 m^2^/g.^31,33,34^

The results obtained for the PL and NC samples align with the observations made by X-ray diffraction (XRD) and Fourier-transform infrared (FTIR) spectroscopy. After stability testing in water, the surface area of the NC sample was reduced to zero, indicating complete dissolution of the structure. There was also a reduction in the initial surface area of the PL sample, from 794 to 594.54 m^2^/g after the test. Previous studies have indicated that reducing the particle size of metal–organic frameworks (MOFs) remains a significant challenge. Manipulating the particle size of ZIF-67 by varying the concentration of CTAB resulted in a decrease in the particle size and a corresponding reduction in the surface area as the surfactant concentration increased. Conversely, ZIF-8 particles synthesized with CTAB exhibited large surface areas for plate-like and nanocube structures, ranging from 1400 to 1600 m^2^/g. As the surfactant concentration increases, a reduction in porosity is reported, which is attributed to the incorporation of CTAB molecules into the ZIF-8 structure, rather than adsorption of CTAB on the crystal surfaces.^22^ In the context of mixed matrix membrane production, the decrease in surface area reduces the number of sites for adsorption of gas molecules and should affect gas separation, reducing the efficiency of mixed matrix membranes. RD and RD-1 particles will benefit from the exposure to water and ethanol during membrane production, but PL and NC efficiency should be reduced. Nevertheless, the prediction of the effect of ZIF-67 stability in mixed matrix membranes is difficult due to possible interactions between imidazolate and zinc with the polymers that form the membranes. Based on surface area alone, it could be stated that the sieve effect of PL and NC should be diminished after exposure to water and methanol.

Analysis of the images obtained by scanning electron microscopy (SEM), shown in Figure 10, revealed notable differences between ZIF-67 RD-1 synthesized at room temperature with methanol and a 1:8 stoichiometry (metal:MeIM) and the RD structure. ZIF-67 RD-1 exhibited a more homogeneous particle distribution with a regular morphology and an average particle size of 300 nm, whereas the RD images showed larger, less aggregated particles, with an average particle size of 400 nm and a size distribution ranging from 180 to 500 nm. These morphological variations are attributed to a combination of factors, including the MeIM ratio, solvent type, changes in the chemical source, and temperature effect in hydrothermal synthesis.^18,47,48^ Previous studies have shown that ZIF-67 crystals prepared under nonhydrothermal conditions were larger than those prepared under hydrothermal conditions. This occurs because the reagents dissolve more completely under hydrothermal conditions, forming numerous crystal nuclei with a rapid growth rate, resulting in smaller crystals.^47,49^

**Figure 10:**
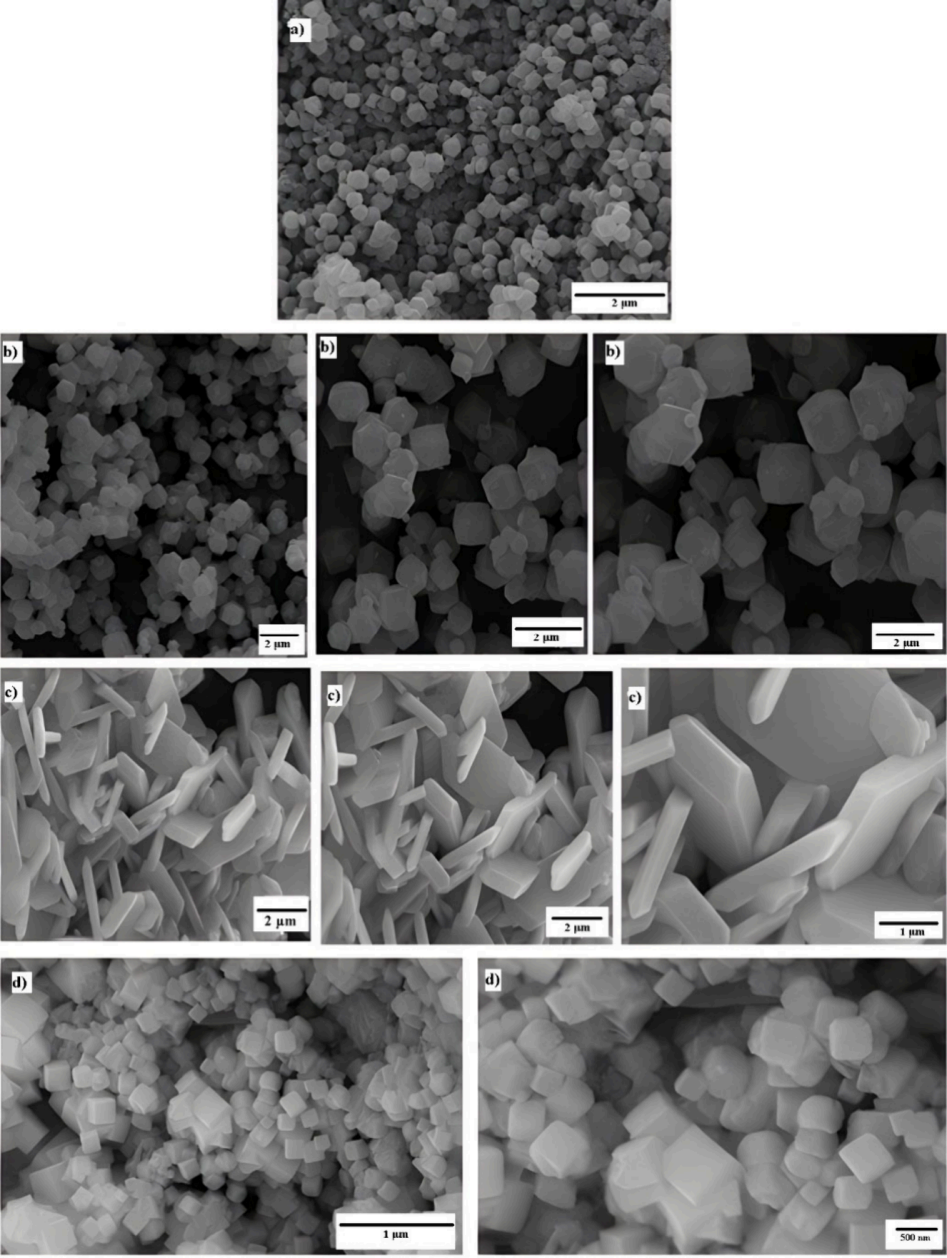
Scanning electron microscopy (SEM) images of the synthesized ZIFs: (a) RD-1, (b) RD, (c) PL, and (d) NC.

Using a CTAB concentration of 0.12 wt %, the synthesized ZIF adopts a plate-like morphology with a smooth surface, with a thickness ranging between 100 and 300 nm. Yang et al.^22^ indicated that RD and nanocube morphologies (0 and 0.08 wt %) exhibit similar particle sizes and shapes both at the initial synthesis stage and after extended synthesis periods, with little or no CTAB added. In contrast, for morphologies such as plates, at higher CTAB concentrations (>0.12 wt %), crystal growth becomes highly dependent on the synthesis kinetics. In plates, there is a more pronounced increase in the lateral dimension of the crystals with synthesis time, resulting in an increase in the aspect ratio. This deceleration of crystal growth upon addition of CTAB can be attributed to the competition between ligands (Hmim) and surfactants (CTAB) for interaction with the chemical source. A significant variation in the size of the nanocubes (NC) synthesized was observed, ranging from 180 to 800 nm, with an average particle size of 600 nm.

## Conclusions

Optimization of the synthesis parameters revealed that the ideal conditions were achieved through the complete dissolution of 2-methylimidazole and CTAB at room temperature, followed by contact with the diluted metal/ligand solution. Vigorous agitation of the solution proved to be essential for obtaining particles with a regular and homogeneous morphology. The introduction of the surfactant CTAB facilitated the formation of micelles, resulting in structures with enhanced crystallinity and a uniform particle size distribution. The synthesis conducted at 140 °C was superior to that at 120 °C, exhibiting a more regular and well-defined morphology.

Hydrothermal stability studies of the hierarchical ZIF-67 particles demonstrated that the RD and RD-1 samples, synthesized without the CTAB surfactant, exhibited greater structural stability compared with the PL and NC samples containing the surfactant. XRD, FTIR, and TGA analyses confirmed that the RD and RD-1 samples retained their crystalline characteristics, displaying superior resistance to degradation and lower water absorption, while the CTAB-containing samples showed an increased susceptibility to hydrolysis. These findings highlight the significance of forming well-defined structures and ensuring the complete removal of the residual surfactant to ensure the hydrothermal stability of ZIF-67.

In conclusion, strict control of the synthesis parameters is essential to obtain ZIF-67 particles with optimized structural and morphological properties, crucial for technological applications. The structural stability and large surface area of the RD-1 and RD samples reinforce their potential in gas separation and CO_2_ capture processes, particularly in membrane-based technologies. This study significantly advances the understanding of the impact of synthesis conditions on the quality of ZIF-67, providing valuable insights into its reliable reproduction and application in industrial and environmental contexts.

## Supplementary Material



## Notes

Influence of mixing sequence on the synthesis of hierarchical ZIF-67 structures (MeIM:H_2_O molar ratio of 120), XRD patterns of ZIF-67 synthesized under varying stirring speeds and temperatures (Figure S1), SEM images of ZIF-67 obtained under different synthesis conditions (120 or 140 °C, low or high stirring rate) (Figure S2), thermogravimetric analysis of ZIF-67 particles, thermal degradation in synthetic air (Table S1), and thermal degradation in a N_2_ atmosphere (Table S2) (PDF)
