# Supplementary material for: Controlling the Hierarchical Morphology of ZIF-67 via Hydrothermal Synthesis: Insights into the Stability for Gas Separation
Source: Langmuir. 2025 Jul 9;41(29):19134–45. doi: 10.1021/acs.langmuir.5c01142 (PMC12312155; doi:10.1021/acs.langmuir.5c01142)
Supplement: Supplementary file 1 [file la5c01142_si_001.pdf]

Supporting Information

Controlling the Hierarchical Morphology of ZIF-67 via Hydrothermal Synthesis: Insights into Stability for Gas Separation

*Paula S. Pacheco [a, *], Daniel Eiras [a], Sônia Faria Zawadzki [b]*

[a] Graduate Program in Materials Science and Engineering *(PIPE/UFPR)*, Federal University of Paraná, Jardim das Américas, Curitiba, PR, Brazil, ZIP: 81530-000.

[b] *Graduate Program in Chemistry (PPGQ/UFPR), Federal University of Paraná, Jardim das Américas, Curitiba, PR, Brazil, ZIP: 81530-000.*

* Corresponding Author: paula.sacchelli@gmail.com

Influence of Mixing Sequence on the Synthesis of Hierarchical ZIF-67 Structures: MeIM/H_2O molar ratio of 120.

Figure S1. XRD curves of ZIF-67 obtained with different stirring speeds of the mixture and synthesis temperature for molar ratios of MeIM/H2O of 120.

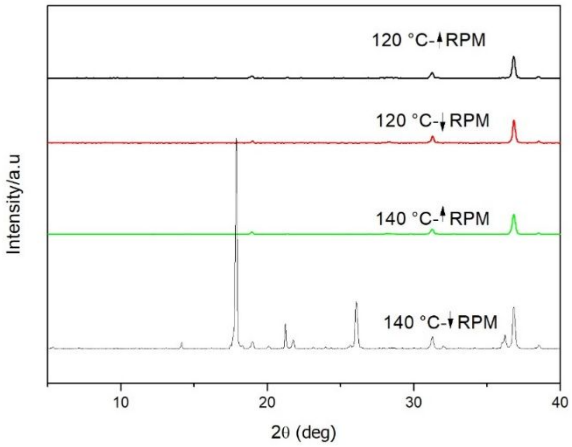

Figure S2. Scanning electron microscopy of the ZIF-67 obtained with different stirring speeds of the mixture and synthesis temperature for molar ratios of MeIM/H2O of 120: a) 120°C and high rotation, b) 120°C and low rotation, c) 140°C and high rotation.

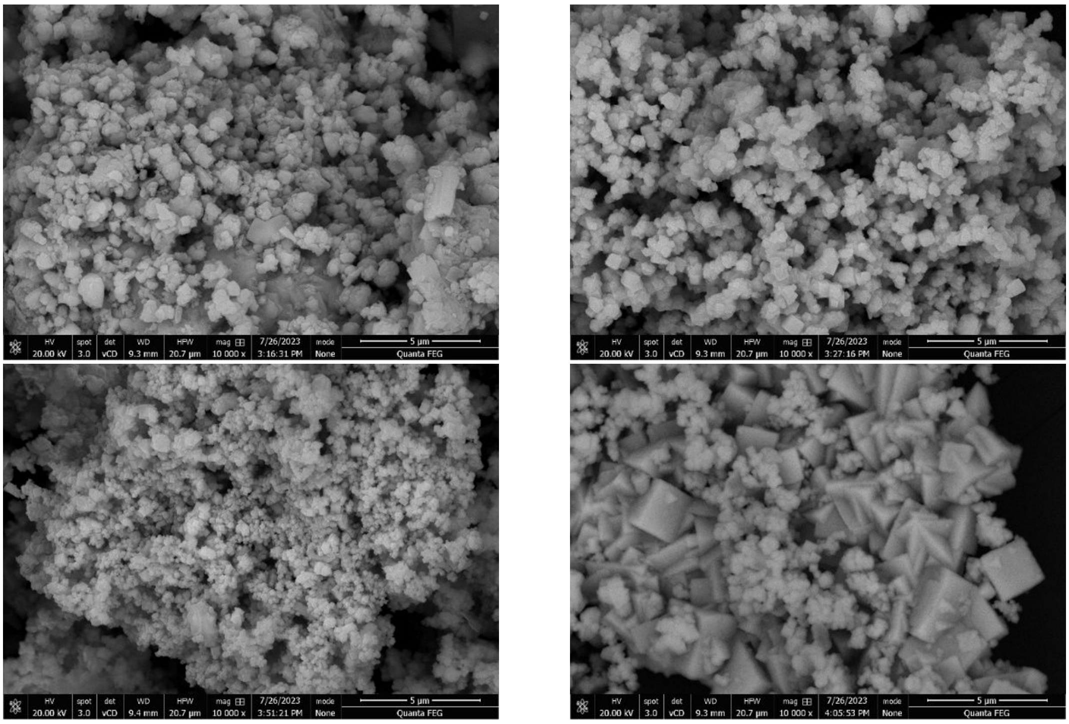

Synthesis of ZIF-67 Particles and Study of Hydrothermal Stability: Thermogravimetric analysis.

Table S1. Thermal Degradation in Synthetic Air

Sample	1st Mass Loss (°C)	2nd Mass Loss (°C)	Weight Loss (%)
RD-1	149–306	390–450	33.7
RD	36–212	360–408	24
PL	-	350–561	35
NC	64–196	250–425	64

Table S2. Thermal Degradation in N_2 Atmosphere.

Sample	1st Mass Loss (°C)	2nd Mass Loss (°C)	Weight Loss (%)
RD-1	156–301	477–625	56
RD	119–215	489–622	45
PL	-	323–551	63
NC	32–148	340–528	59
